# Supplementary figures and images for: Are Different Populations Fairly Represented in Single-Cell Omic Atlases?
Source: bioRxiv. 2026 May 20:2025.10.01.677375. Preprint. [Version 2] doi: 10.1101/2025.10.01.677375 (PMC13228520; doi:10.1101/2025.10.01.677375)

**A**

### HCA: Tissue Type Distribution

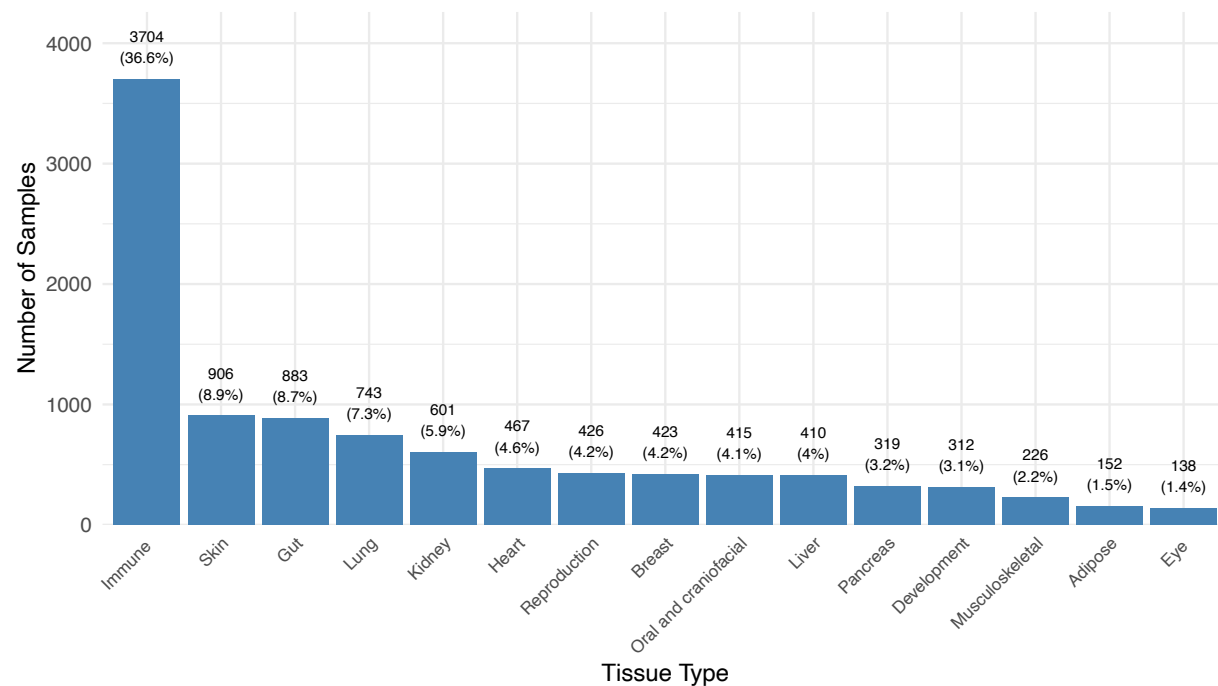**B**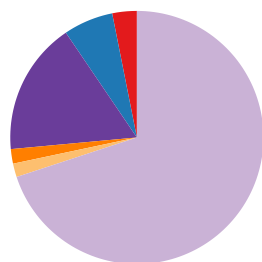

### Ancestry

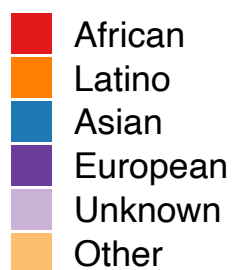**C**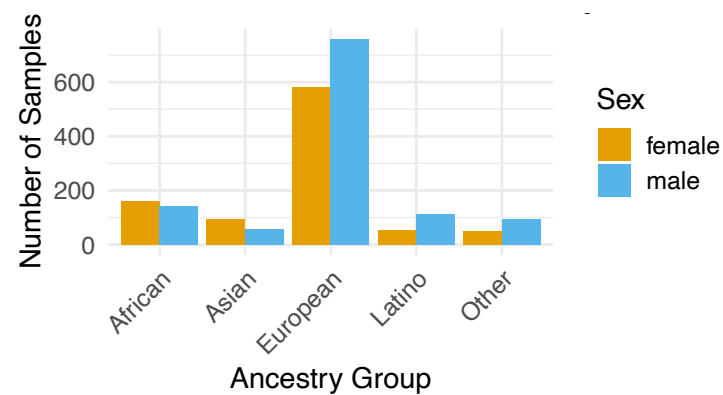

**A** HTAN: Cancer Type Distribution

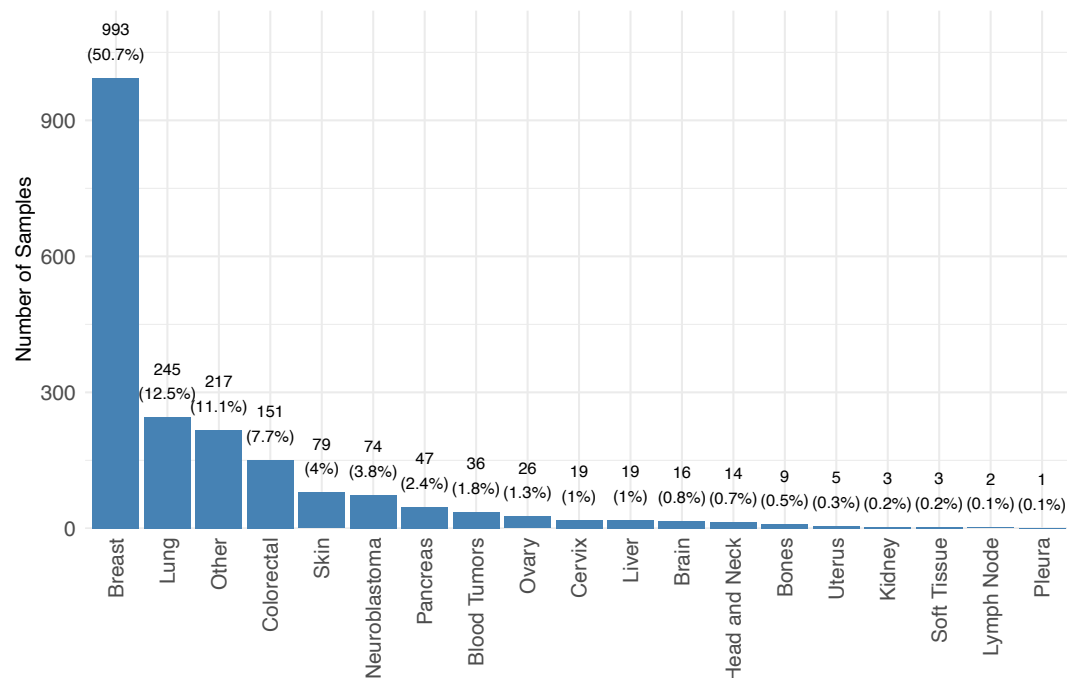

**B**

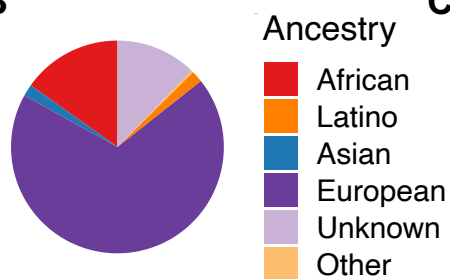

**C**

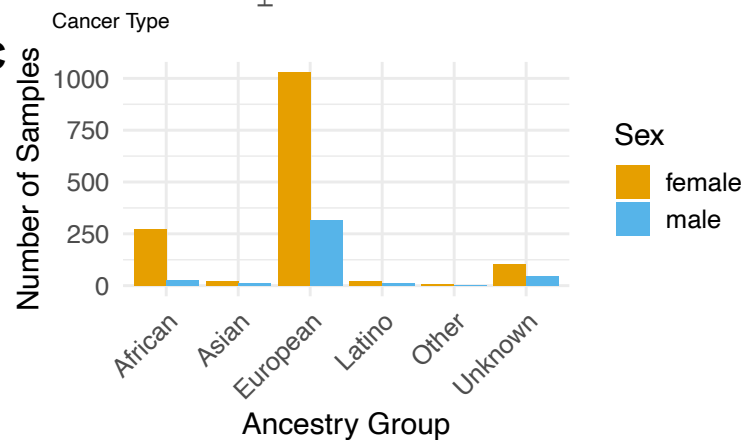

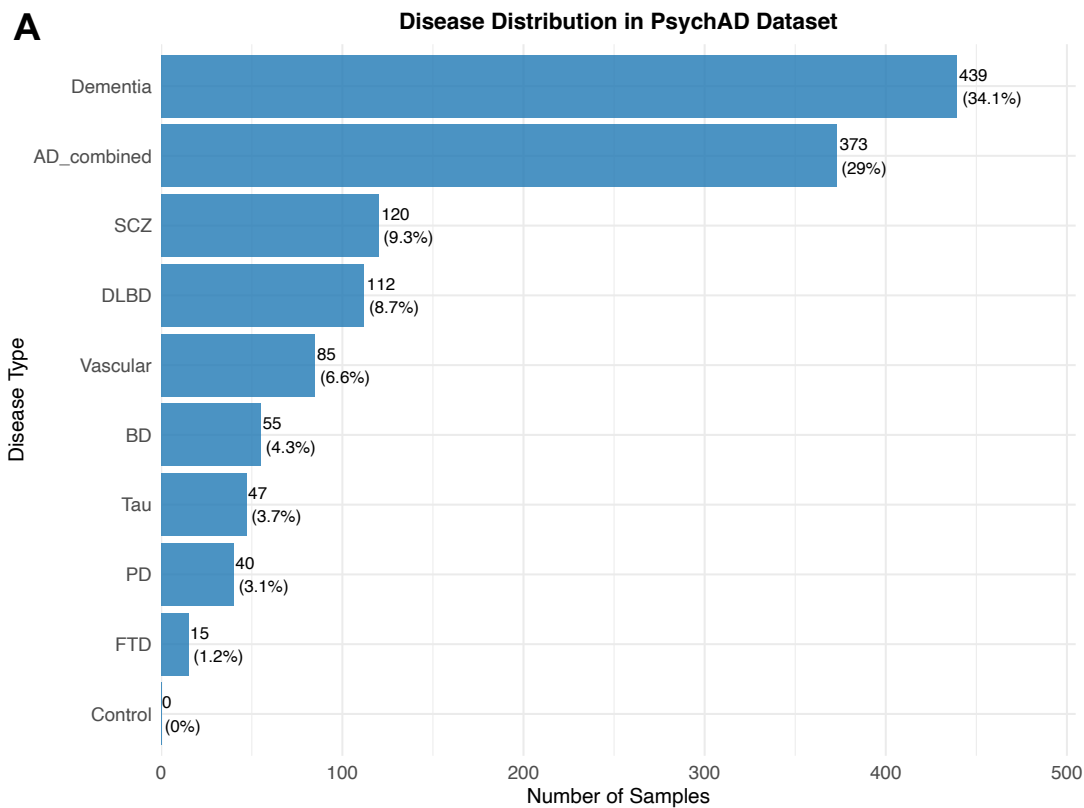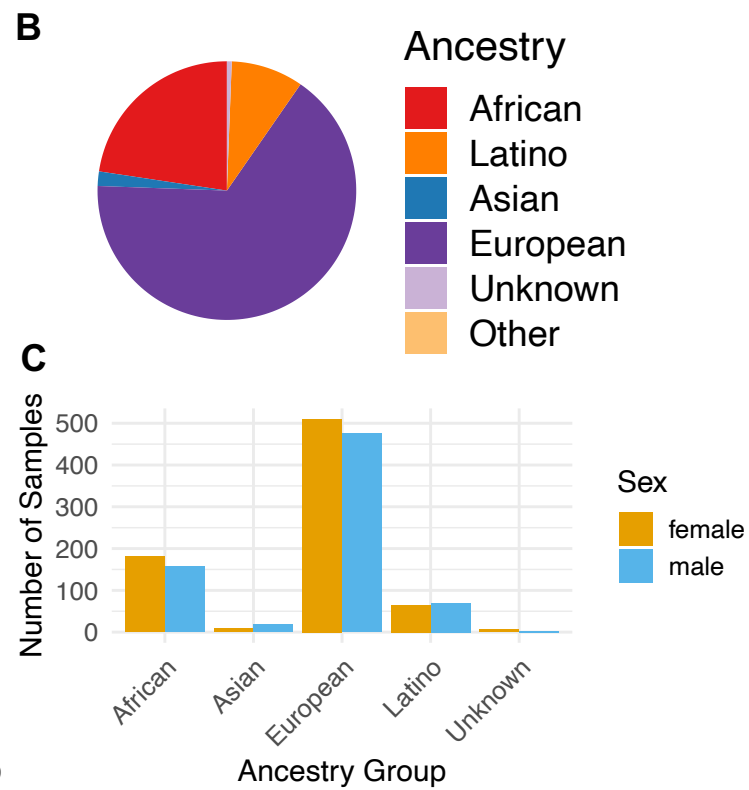

Supplement: Supplement 1 — Supplementary Figure 1. Human Cell Atlas (HCA) dataset composition (A) Barplot of tissue type distribution across HCA, showing sample counts for tissue categories. (B) Pie chart of ancestry distribution across all HCA samples, showing proportions of African, Asian, European, Latino, Other, and Unknown ancestry. (C) Stacked barplot of sex distribution by ancestry group in HCA. Supplementary Figure 2. Human Tumor Atlas Network (HTAN) dataset composition (A) Barplot of cancer type distribution across HTAN, showing sample counts for cancer types. (B) Pie chart of ancestry distribution across HTAN, showing proportions of African, Asian, European, Latino, Other, and Unknown ancestry. (C) Stacked barplot of sex distribution by ancestry group in HTAN. Supplementary Figure 3. PsychAD dataset composition (A) Barplot of disease type distribution across PsychAD. (B) Pie chart of ancestry distribution across all PsychAD samples, showing proportions of African, Asian, European, Latino, Other, and Unknown ancestry. (C) Stacked barplot of sex distribution by ancestry group in PsychAD. Supplementary Figure 4. PsychAD dataset ancestry and sex composition, , shown in counts and percentages within each disease type Supplementary Table 1. Human Cell Atlas (HCA) dataset ancestry and sex composition, shown in counts and percentages within each tissue type Supplementary Table 2. Robustness analyses of ancestry representation in the Human Cell Atlas (HCA) dataset Supplementary Table 3. Human Tumor Atlas Network (HTAN) ancestry and sex composition, shown in counts and percentages within each cancer type [file media-1.pdf]
